# A novel hybrid-maximum neural network in stereo-matching process

**DOI:** 10.1007/s00521-012-1202-0

**Published:** 2012-10-12

**Authors:** Łukasz Laskowski

**Affiliations:** Department of Computer Engineering, Czestochowa University of Technology, Al. A.K. 36, 42-200 Czestochowa, Poland

**Keywords:** Hopfield, Stereovision, Neural network, Hybrid network, Depth analysis

## Abstract

In the present paper, the completely innovative architecture of artificial neural network based on Hopfield structure for solving a stereo-matching problem—hybrid neural network, consisting of the classical analog Hopfield neural network and the Maximum Neural Network—is described. The application of this kind of structure as a part of assistive device for visually impaired individuals is considered. The role of the analog Hopfield network is to find the attraction area of the global minimum, whereas Maximum Neural Network is finding accurate location of this minimum. The network presented here is characterized by an extremely high rate of work performance with the same accuracy as a classical Hopfield-like network, which makes it possible to use this kind of structure as a part of systems working in real time. The network considered here underwent experimental tests with the use of real stereo pictures as well as simulated stereo images. This enables error calculation and direct comparison with the classic analog Hopfield neural network as well as other networks proposed in the literature.

## Introduction

**Fig. 1:**
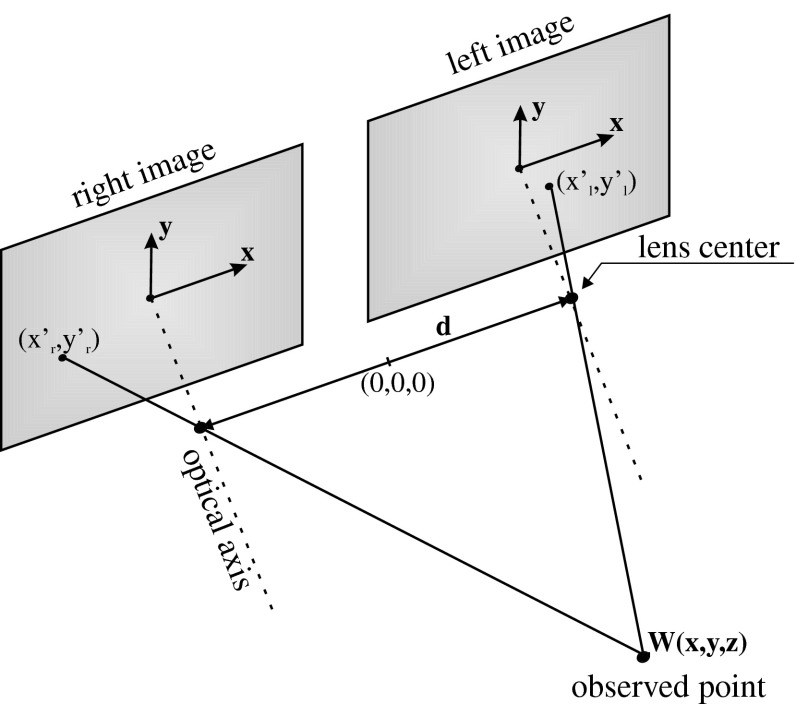
The configuration of a parallel stereovision system

The use of stereovision is a natural way of determining the distance by the humans. This idea is not new. The simplified model of human sight can be presented as two parallel cameras, and this model (named parallel stereovision system) will be considered in this. The above-mentioned systems have been widely applied in numerous fields such as cartography, psychology, neurophysiology, visually impaired support, a vehicle driving support, robots navigation, and a lot of others. This wide application of the stereovision systems is due to its unquestionable advantages: do not emit any radiation, like microwave, any physical contact with environment is necessary, like in the case of white cane, and is easy to apply. Figure 1 illustrates the geometry of a parallel stereovision system [1]. As can be seen, the system is composed of two cameras. In a parallel stereovision, the optical axes of cameras are located parallelly and perpendicularly to the baseline, connecting the centers of the cameras. As can be clearly seen, the image of the observed point *W* with coordinates (*x*, *y*, *z*) has different positions in planes of left and right cameras. It is easy to notice that the difference in positions of images of point W is the smaller, the further point *W* is located from the point of (0,0,0) (point of reference (0,0,0) is located between lens centers). The expressions on real coordinates can be written as (1).

1 $$ \left\{\begin{array}{l} x = \frac{d(x^{\prime}_l-x^{\prime}_r)}{2(x^{\prime}_l-x^{\prime}_r)} \\ y = \frac{d(y^{\prime}_l-y^{\prime}_r)}{2(x^{\prime}_l-x^{\prime}_r)} \\ z = \frac{df}{x^{\prime}_l-x^{\prime}_r}\end{array}\right. $$

As can be clearly seen, the distance to the observed point is inversely proportional to the difference in position of the image of this point on plane left and right (disparity). While looking at the Eq. (1), it seems possible to determine the distance to each point at an observed scene (depth analysis [2–4]). The problem seems to be trivial and it is trivial up to a point. The real scene contains large numbers of points. The main problem lies in finding the corresponding points in left and right pictures. Finding the points in the left and the right images which correspond to the same physical point in space is called the stereo correspondence problem. The complexity of the correspondence problem depends on the complexity of the scene. This is a very difficult problem to solve. In theory, general solution may not exist, given the ambiguity which results from textureless regions, occlusion, specularities, and the like. From a computational standpoint, trying to match each of the pixels in one image to each of the pixels in the other image is extremely difficult, given the massive number of comparisons. There are constraints and schemes that can help reduce the number of false matches, but many unsolved problems still exist in stereo matching [5–7]. The main problems include the following: occlusion, discontinuity of depth, discontinuity of periphery, regularity, and repetitivity. For this reason, the stereo-matching problem is one of the most complex problems in the computer vision, and it is of vital importance to search a new and efficient way to solve this task.

This paper proposes using a novel neural structure based on Hopfield neural network—Hybrid-Maximum Neural Network (HMNN). The short introduction to this kind of structure can be found in [8]. This design is novel and had not been used in the literature before. This kind of structure can also be used for other optimization problems. The main advantage of HMNN is its speed and accuracy (the number of network’s iteration decreases at least down to 0,7 of epochs number of the analog Hopfield-like network). Another advantage is a simplification of energy function (which caused the decrease in the number of local energy minima) and automatic upkeep of uniqueness in certain optimization problems (N-queens, stereo correspondence). Also, the energy function for stereo correspondence problem presented in the article is novel. The formula for the energy of network, proposed by the author, seems much more appropriate for stereo matching, than other formulas available in the literature (see Sect. 3).

The aim of this paper is to describe and test the Hopfield neural network—Hybrid-Maximum Neural Network in stereo-matching problem. The architecture, energy functions (the energy of network forms changes in the course of the network’s work), and working algorithm are presented here. Also, the tests results, based on testing real images, are included in comparison with some other Hopfield-based neural networks' working results. The accuracy of the solution and the efficiency are discussed. The depth maps, obtained by each investigated network, are also shown in the paper.

### Background

In the literature, one can find a few types of algorithms for solving the stereo correspondence problems [9]. The main problems are as follows:

- *Feature based* algorithms [10, 11] which establish correspondences between some selected features, extracted from the images, such as edge pixels, line segments, or curves [12–19].
- *Phase based* algorithms based on the Fourier phase information which can be considered as a sort of gradient-based optical flow method, with time derivative approximated by the difference between the left and right Fourier phase images [20–22]. This idea became really applicable with the introduction of localized frequency filters called Gabor filters. This method computes the convolution between Gabor kernels and the left and right image parts. In order not to get trapped in some local minimum, the hierarchical methods were used here [23–25].
- *Energy based* algorithms This kind of approach is based on the minimization of energy function representing a given problem (in this case, stereo-matching problem of course), [26–29]. This one seems to be the most universal, powerful, and developed out of all the above-mentioned methods.
- *Area-based* algorithms [30–32] are based on the division of the images into subareas, which are fitted. These methods are well adapted for relatively textured areas. However, they generally assume that the observed scene is locally fronto-parallel, which causes problems for slanted surfaces and, in particular, those which are near the occluding contours of the objects. Finally, the matching process does not take into account the edge information, which is actually very important and should be used in order to get the reliable and accurate dense maps.

Nowadays, the algorithms in their original forms, as described above, are rarely used and their application is limited only to very basic problems. Scientists are trying to merge different types of solution in order to take as many advantages from all types of algorithms as possible and avoid disadvantages. A very interesting development of the area-based algorithm was proposed by Sun and coworkers [33–35]. The authors developed stereo-matching algorithm that produces a dense disparity (depth) map by means of cross-correlation, rectangular subregioning (RSR), and 3D maximum-surface techniques in a coarse-to-fine (pyramid) scheme. The correlation is achieved by means of the box filtering technique and by segmenting the stereo images into rectangular subimages at different levels of the pyramid. The disparity map for the stereo images is found in the 3D correlation coefficient volume by obtaining the global 3D maximum surface, rather than simply choosing the position that gives the local maximum correlation coefficient value for each pixel. The 3D maximum surface is obtained by means of a two-stage dynamic programming (TSDP) technique. This method seems very promising, but rectangular segmenting can generate false fitting.

The use of energy method to weekly calibrated stereo pictures was presented by Alvarez et al. [9]. At first, the authors found a simplified expression of the disparity that allows us to estimate it from a stereo pair of images by means of an energy minimization approach, assuming that the epipolar geometry is known, and they included this information in the energy model. The energy function is minimized by means of a gradient descent method. The results of the experiments are very promising, but gradient minimization could work slowly and there is a possibility of being trapped in local minimum of energy.

The energy can be also minimized by using Hopfield-like neural nets [36–40]. The ability of the Hopfield network to solve the optimization problems relies on its steepest descent dynamics and guaranteed convergence to local minima of the energy landscape. The advantage of Hopfield-like neural networks over the gradient minimization methods depends on fast operation. This computational model is massively parallel, which is very important as far as real-time working systems are concerned. This kind of system was used in stereo-matching problem [41–43]. In [44] and [45], the authors described a driving support system based on stereoscopy and Hopfield-like analog neural nets. Unfortunately, the authors did not include any clear depth map that could result from the application of their system, so it is difficult to estimate the efficiency of the system. Also, the form of energy function is unclear. It is worth noting that the authors decreased the calculation time by the elimination of a certain number of neurons. This author has tested the use of continuous Hopfield-based neural network with the energy function worked out by himself. Results of simulation were fairly good, which was presented in Sect. 3. The only disadvantage was the low speed of network’s computing. A similar system for reconstruction of the third dimension of scene from stereo pictures with the use of analog Hopfield-like neural nets was described in [45]. Also, in this case, the authors did not include any reliable depth map that could allow the judgement of efficiency of the algorithm. Discrete asynchronous Hopfield neural net, used for solving stereo-matching problem, was described in [46] by Sun and al. The authors of [46] presented very good results of stereo matching by using a Hopfield-like net. The author of this publication tried to repeat simulations according to algorithm presented in [47]. Unfortunately, the attempt to repeat their results came to grief, which was predictable. The results of these simulations have been shown in Sect. 3. As far as discreet dynamics Hopfield nets to optimization problems are concerned, the networks with continuous activation function should be preferably used. In [48], the authors obtained good results of liver stereoscopic visualization, but it should be mentioned that those results were achieved for moderate complex pictures: Only selected (characteristic) points on the liver’s pictures were matched. The author tried using the method described in [48] neural structure (discreet Hopfield-like network with continuous activation’s function), but the results were disappointing, which has been presented in Sect. 3.

Despite some imperfections of Hopfield’s network’s work performance, quoted here, the author claims that such structures are the best way of solving the stereo-matching problem. Hopfield’s-like structures enable to express the problem holistically distinct from classical algorithms, which focus on one point. The ability of parallel working of each neuron gives the opportunity for preparing a device working extremely fast without losing its accuracy. It is very important in systems demanding the real-time action.

Looking at the state-of-the-art-of stereovision matching with the use of Hopfield-like networks, one can have an impression that this domain has been well explored. However, none of the networks, described in the above-mentioned articles, work in a way that is efficient enough. The author tried to use the above-mentioned solution in the stereo-matching process. Unfortunately, the application of the above-mentioned solution to the stereo-matching process each time resulted in the number of errors of the network’s work performance exceeding 20 % (the way of error calculation and experimental conditions were described in Sect. 3), which practically eliminates those methods of solving the stereo-matching problem. The subject presented here is so wide and complicated that it is still possible to improve the efficiency of such systems, to work out better architecture of nets and decrease the number of errors of the stereo-matching process.

As a result of a thorough analysis of neural solution of stereo-matching problem, presented in the literature, the author came to a conclusion that the main problem is trapping the network in local minima of energy. It is not possible to write an energy function with only one global minimum. In this case, a discrete Hopfield-like neural network fails. Much better results can be reached with the help of an analog Hopfield-like neural network. The disadvantage of this kind of nets is the time of minimum’s reaching. A lot of stereovision systems demand real-time working. The author made an attempt to combine the *f* efficiency of the analog Hopfield-like network with an extremely high rate of work performance in case of particular kind of discrete Hopfield-like network. Both the analog Hopfield network and the Maximum Hopfield-based network had been used in optimization problems before, but the Network being the combination of both types of network had never been used before. Also, the energy functions forms, with the use of neural structures creating a hybrid network, have been worked out by the author. The author’s research has resulted in a completely innovative architecture of a network which solves the stereo-matching problem: a hybrid neural network consisting of the classical analog Hopfield neural network and the Maximum Neural Network—the Hybrid-Maximum Neural Network (HMNN). Due to the use of these two types of networks, described here, the structure is working much faster than the classical Hopfield net and its accuracy is not worse. The efficiency of HMNN was confirmed in tests on real and simulated pictures (which allowed the error calculation). Following comprehensive study, there is no doubt that the results obtained with the help of the HMNN are the same as for continuous Hopfield-like network, but the time of reaching them was much shorter than for continuous structure.

## Architecture

The stereo-matching problem can be referred to as an optimization task where the energy function, which represents the constraints on the solution, is to be minimized. The optimization problem then can be solved by means of the Hopfield neural network [49, 50]. The most accurate solution can be obtained by analog network.

The disadvantage of the analog Hopfield neural network is its long time of computation. The speed of operation is very important as far as the target system is concerned—it should work in real time. Much faster is a Maximum Neural Network [51]. The additional advantage of a maximum network is that stereo matching is reciprocally unique thanks to network’s architecture. Unfortunately, the accuracy of solution found by the Maximum Neural Network is much worse than in the case of analog Hopfield-like network.

**Fig. 2:**
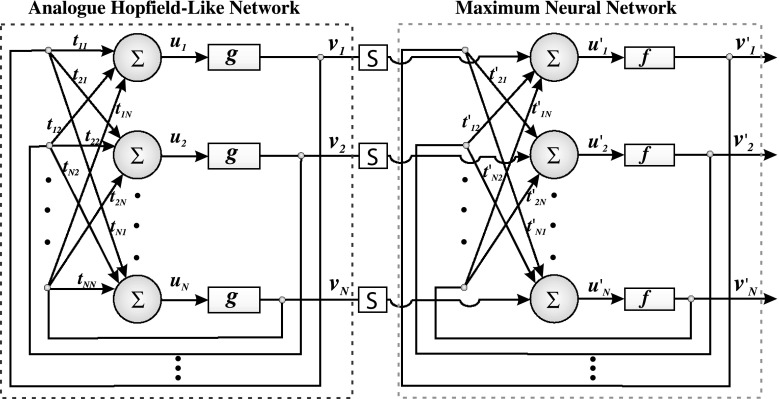
An architecture of Hybrid-Maximum Neural Network

However, it is possible to combine the precision of work performance of the analog Hopfield network with the speed of maximum neural network’s operation. The hybrid neural network presented here contains both the analog Hopfield network and the maximum neural network. The architecture of the neural network described here is shown in Fig. 2.

In the first stage of HMNN’s work performance, the analog Hopfield neural network is looking for the attraction area of the global minimum. Having found the attraction area of global minimum, the network is switched to its maximum mode thanks to the block of switching function *S* (see Fig. 2).The switching follows a given number of iteration (determined empirically). The switching function can be defined as follows:

2 $$ f(v_{ij},it) = \left\{\begin{array}{ll}v_{ij}&\hbox{for}\;it=it_{\rm max}\\ 0&\hbox{ for }\;it\neq it_{\rm max}\end{array} \right., $$

where *it* is an iteration number, and *it*_max_ is assumed maximum number of iteration in continuous mode. In maximum mode, the network is quickly evaluating toward the global minimum and the term of uniqueness is kept automatically thanks to the maximum activation function (all terms are described in the further section).

### The analog Hopfield neural network for stereo-matching problem

In the first stage, HMNN is working in continuous mode, in the same way as continuous Hopfield-like neural net [49, 50].

**Fig. 3:**
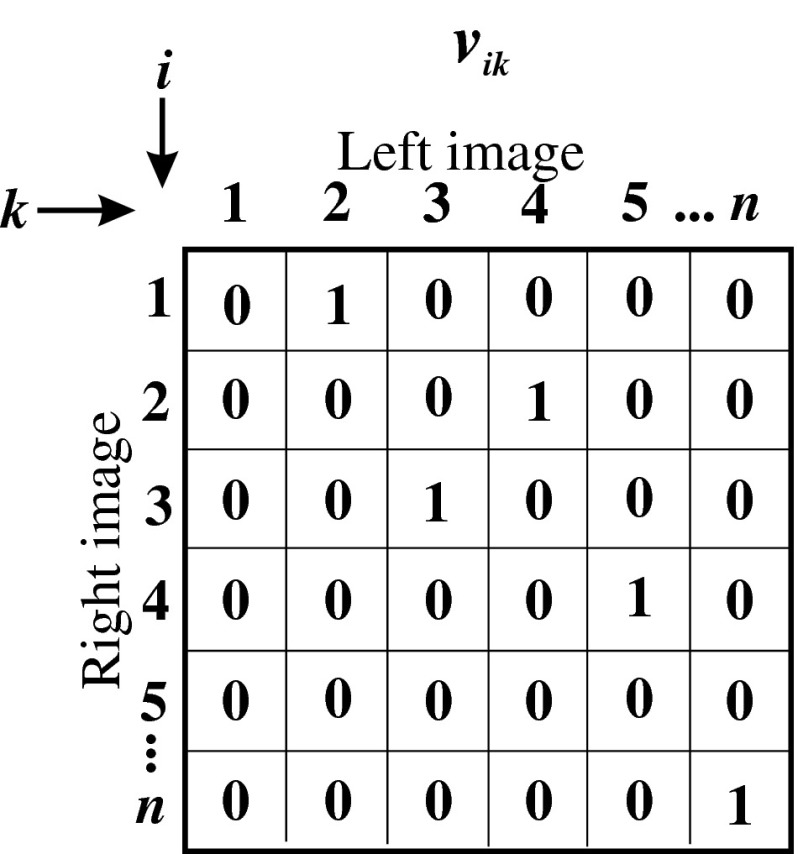
The fitting matrix, representing one epipolar line, for Hopfield-like neural network dedicated to solving stereo-matching problem

Assuming that both stereo images have the length of *n*, the proposed network consists of *n* × *n* neurons for one epipolar line in an image. For pictures with the height equal *h*, it is easy to note that the target system will consist of *h* networks working in a parallel way—each network will realize a stereo-matching problem for one epipolar line. Each neuron neu_*ik*_ is responsible for fitting *i*-point in right image to *k*-point in left image. The higher the external potential of neu_*ik*_, the better the fitting of points becomes. In the final configuration, only for corresponding points *i* in right image to *k* in left image, potential *neu*_*ik*_ will equal 1, and for the rest of the points, the external potential of neurons will equal 0. It is very convenient to represent neurons as a matrix, named fitting matrix (FM), as shown in Fig. 3.

As can be easily concluded, the one in FM means fitting of points, and the values between zero and one (for continuous activation function, used here) can be interpreted as probability of stereo matching of points.

The Hopfield computational energy *H* associated with the network state *v* is given by (3)

3 $$ H(t)=-\frac{1}{2}\sum_{i}\sum_{k}\sum_{j}\sum_{l}t_{ij} v_{ik} v_{jl} - \sum_{i}\sum_{k}I_{ik} v_{ik}+\frac{1}{\tau} \sum_{i}\sum_{k}C(v_{ik}), $$

where *t* is the weight matrix, and *I*_*i*_ is the firing threshold of neuron *i*, *v*_*i*_ being the external state of neuron *i*. In the Eq. 3, *C* is expressed by (4)

4 $$ C(x)=\int\limits_{\frac{1}{2}}^{x}g^{-1}(\eta) d\eta . $$

In the Eq. (4), τ is a positive constant (interpreted as neuron relaxation time), and *g* is a continuous activation function with *v*_*i*_(*t*) = *g*(*u*_*i*_(*t*), *u*_*i*_ is internal potential of neuron *i*. In the present work, *g* is a sigmoidal function, expressed by (5)

5 $$ g(x)=\frac{1}{1+e^{-\alpha x}}, $$

where the value of α adjusts the slope of the sigmoidal curve. In the present work, α = 50. It is worth noting that for the high value of α, (3) can be simplified to the following expression:

6 $$ H(t)=-\frac{1}{2}\sum_{i}\sum_{k}\sum_{j}\sum_{l}t_{ik,jl} v_{ik} v_{jl} - \sum_{i}\sum_{k}I_{ik} v_{ik} . $$

The equation of the motion of the Hopfield model is given as follows:

7 $$ \frac{du_{ik}}{dt}=\sum_{j}\sum_{l}t_{ik,jl}v_{jl}+I_{ik}-\frac{u_{ik}}{\tau}. $$

Because of using of software realization of the network by CPU, instead of mapping of each neuron into separate units connected in Hopfield structure, the equation of dynamics for Hopfield network must be discretized by means of a numerical method. In this case, the Euler discretization was used [52–55]:

8 $$ u_{ik}(t+1)=u_{ik}(t)+\Updelta t \left(\sum_{j}\sum_{l}t_{ik,jl}v_{jl}(t)+I_{ik}-\frac{u_{ik}(t)}{\tau}\right). $$

In (8), $\Updelta t$ is time step. In the presented design, the value of 10^−3^ has been chosen, which has been determined to be small enough for the Euler rule to provide enough accuracy.

#### The energy function

In the method presented here, crucial for work performance of both the analog Hopfield-like network and the maximum network is the energy’s function. Hopfield neural network is the structure inspired by the spin-glass and, similar to this system, has a property of energy minimization [56–58]. Network proceeds minimization of this function until the minimum is found. This means that the solution to the problem has been found. The energy function is very similar for both types of component networks. Minimization of the energy function must secure the following criteria:

1. For couples of correlated points (*i*, *k*) and (*j*, *l*) in given epipolar line, where *i* and *j* are numbers of point in right image, *k* and *l* are numbers of point in left image, correlation coefficient *C*_*ik*,*jl*_ should have as high value as possible—term of *Correlation*;
2. Assigning must be reciprocally unique—term of *Uniqueness*;
3. The sequence of assigning in areas must be kept—term of *Area Sequence*;
4. The continuity of assigning in areas must be kept—term of *Continuity*;
5. The global sequence of assigning must be kept—term of *Global Sequence*;

A stereo-matching problem is a multicriterion (vectoral) problem [59, 60]. The energy is treated here as vector and each criterion, mentioned above, is represented by a component. In this case, the energy can be written as:

9 $$ \overrightarrow{{\hbox{IEF}}}={\hbox{IEF}}(\overrightarrow{E_1}, \overrightarrow{E_2}, \overrightarrow{E_3}, \overrightarrow{E_4}, \overrightarrow{E_5}) . $$

The main problem in the case of vectoral problem is scalarization:

10 $$ {\hbox{IEF}}=aE_1+bE_2+cE_3+dE_4+eE_5 . $$

where *a*, *b*, *c*, *d*, *e* are weight coefficients of each energy component. The problem of scalarization reduces finding weight coefficients in energy expression (10). The problem is not trivial, not always are the values of energy components known, so it is very difficult to find their hierarchy of importance. In this case, coefficients were found in empirical way, by means of testing images. Knowing the depth map for testing pictures, the weight coefficients were finely tuned toward the error minimization.

The energy components, in the form proposed by the author, are described below.

*The term of Correlation* can be written as the following equation:

11 $$ E_1=-\sum_i\sum_k\sum_j\sum_l C_{ik,jl}v_{ik}v_{vjl} , $$

where *C*_*ik*,*jl*_ is correlation coefficient for points *i* in right image to *k* in left image, and *j* in right image to *l* in left image. The coefficients are calculated according to the following equation:

12 $$ \begin{aligned} C_{ik,jl}=&\sum\nolimits_{x,y=-m}^m \left(\|I^L(i+x,h+y)-I^R(k+x,h+y)\|\right.\\ & +\left.\sum\nolimits_f\|F_{(l)}^L(i+x,h+y)-F_{(l)}^R(k+x,h+y)\|\right)+\\ &\sum\nolimits_{x,y=-m}^m \left(\|I^L(j+x,h+y)-I^R(l+x,h+y)\|\right.\\ & +\left.\sum\nolimits_f\|F_{(l)}^L(j+x,h+y)-F_{(l)}^R(l+x,h+y)\|\right)\\ \end{aligned}, $$

where *I* ^*L*^(*i*, *h*) and *I* ^*R*^(*i*, *h*) mean intensity of left and right image in the point of (*i*, *h*), *F*_(*f*)_^*R*^(*i*, *h*) and *F*_(*f*)_^*L*^(*i*, *h*) is *f*-feature of (*i*, *h*) point in right and left image. The index *m* determines the area of region surrounded by points (the area of regions was assumed as 9, so *m* = 4).

It is easy to note that the value of this term will be the lowest when the high value of potentials *v*_*ik*_ and *v*_*vjl*_ corresponds to the high value of correlation coefficient (note sign of "minus" before the expression).

*The term of uniqueness* can be reduced on the condition of the presence of one high potential (equal one) at the most in every row and, every row and column of FM [61–63]—see Fig. 3. This term can be written as:

13 $$ E_2=\sum_i\sum_k\sum_{l\neq k} v_{ik}v_{jl}+\sum_i\sum_k\sum_{j\neq i} v_{ik}v_{jl} . $$

The foregoing term reaches the minimum which equals zero if and only if in each column and each row, one high potential is present at the most. The lack of high potential in a row or in a column means lack of matching, which is normal in the case of stereo-matching problem (it can come from occlusion and discontinuity of periphery).

*The term of area sequence* can be formulated as follows: *for points i and i+1 belonging to the same areas, if the i point in right image was matched to the k point in left (reference) image (the state of neuron* *neu*_*ik*_ *is high), then to the point i+1 in right image there can be assigned l point only if* *l* ≥ *k*. The area is defined as a part of image between the edges (determined before by the edge detector). This can be expressed as the following equation:

14 $$ E_3=\sum_i\sum_k\sum_{l\leq k} v_{ik}v_{(i+1)l}\sigma_{i,i+1} , $$

where σ_*i*,*j*_ is a term determining whether the points i and j in image belong to the same areas. This can be written as:

15 $$ \sigma_{i,j}= \left\{\begin{array}{ll} 1&\hbox{ if points }i\hbox{ and }j\hbox{ belongs to the same areas} \\ 0&\hbox{ otherwise}\end{array}\right.. $$

**Fig. 4:**
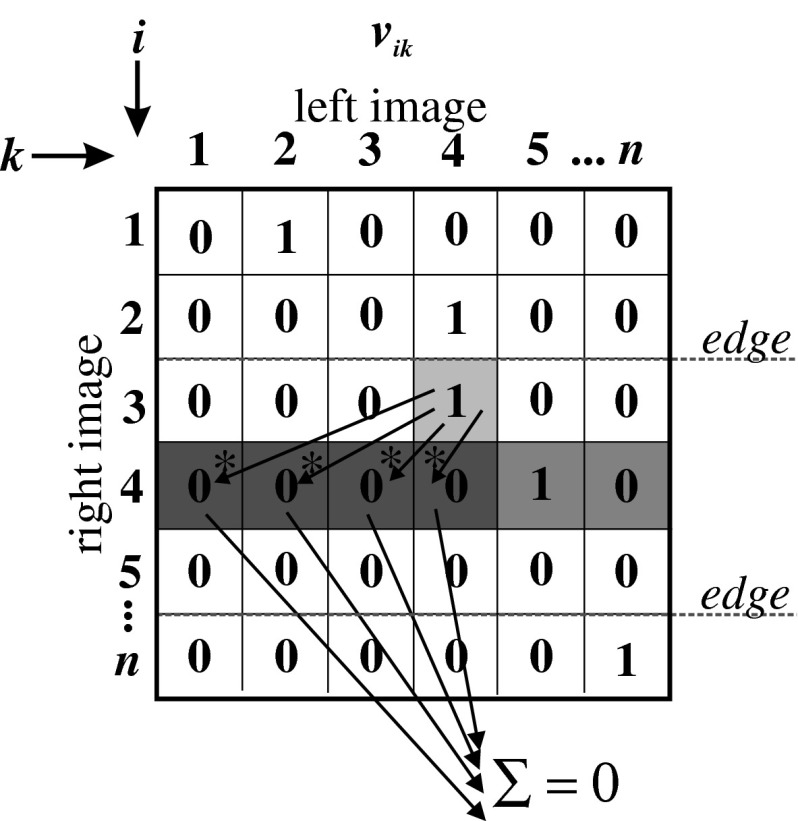
Graphical interpretation of the term of area sequence

As can be easily noticed, the minimum of this term equals zero. A graphical interpretation of this term was shown in Fig. 4.

*The term of depth continuity* can be written in the following way: *if neighbouring points i and j in right image belong to the same areas, the difference in disparity for these points should be as low as possible*. But such formulation can prefer planes perpendicular to optical axe. It is possible yet assumption permissible the angle between object’s planes and optical axis. The term of continuity can be expressed as an equation:

16 $$ E_4=\sum_i\sum_k\sum_{l} v_{ik}v_{(i+1)l}\sigma_{i,i+1} \xi_{ik,jl}, $$

where:

17 $$ \xi_{ik,jl}= \left\{ \begin{array}{ll} ((l-j)-(k-i))-a &\hbox{ for }\;l>k\\ n &\hbox{ for }\;l\leq k \\ \end{array}.\right. $$

**Fig. 5:**
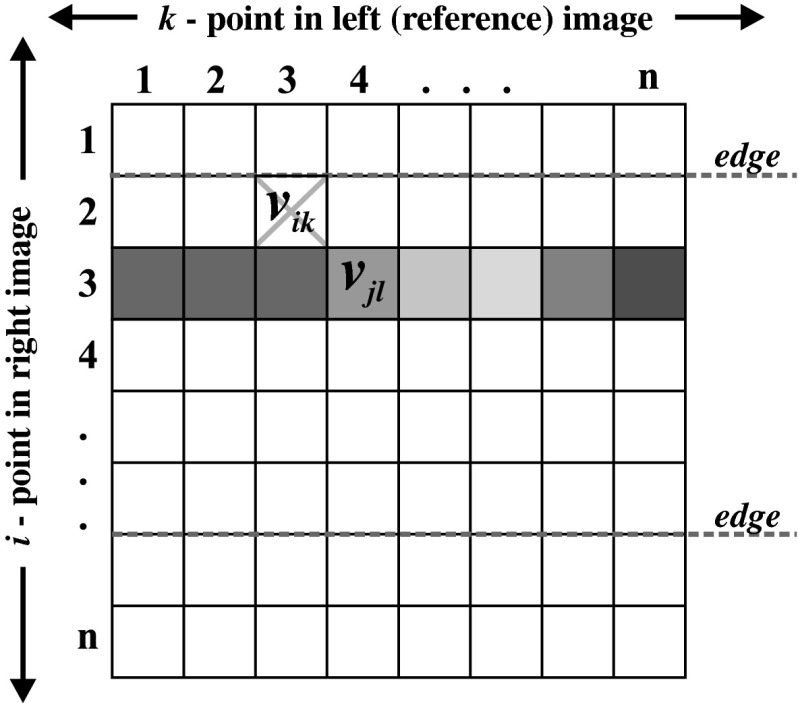
A graphical interpretation of the term of depth continuity. *a* = 2 was assumed. As the green cross pair of assigned points was marked (*v*_*ik*_ = 1). At fields, marked in a red color, high potential is not desirable. At fields, marked in a green color, high potential will cause the decrease in energy value. The fields marked in a blue color are neutral

In (17), *n* is set to a positive number determining energy increase if the term is not kept *a* and to determine an acceptable angle between objects plane and optical axe. This term was depicted in Fig. 5. Thanks to the use of parameters *a* and *n*, there is a possibility to "tune" this term empirically. While looking at the Eq. (17) and Fig. 5, one can notice that if the point was matched to point *k*, for next point *j,* the most favorable matching, from energetic point of view, is next to *k* point *l*. For this layout of matching point, the energy of the term *E*_4_ is zero (with disregarding the *a* parameter). This means that the matched points creating the surface are perpendicular to the optical axis. Thanks to the use of a parameter of *a*, the energy of *E*_4_ can be below zero for matching points not only next to the *k* position. So, the occurrence of surfaces not perpendicular to the optical axis is possible. The parameter *a* can be treated as a shift of possible matching respecting the position *k* (see: Fig. 5).

**Fig. 6:**
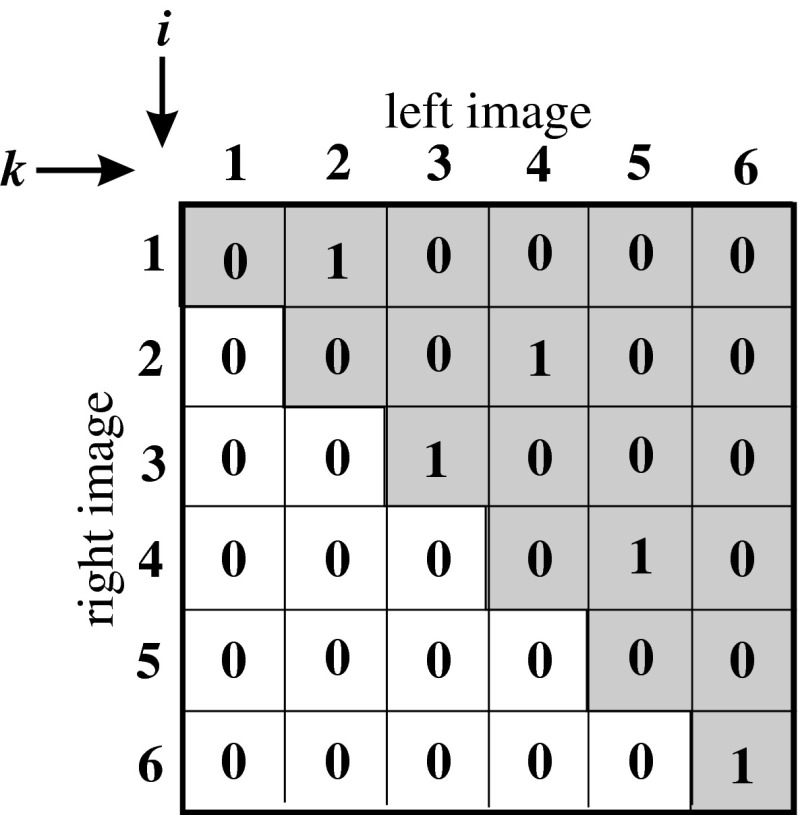
Graphical interpretation of the term of global sequence. Only neurons on fields marked* gray* can evolve. The remainder of neurons is excluded from the network

*The term of global sequence* stems from the direction of displacement of the reference camera in relation to the considered camera. Assuming that right image’s depth map is determinating, the left image is a reference. The point *i* from right image can be matched to point *k* from left image only if *k* > *i*— the image of point *i* cannot occur on position prior to point *i*. This reduces to a triangulation of the FM. It is not necessary for this term to respect this term in energy function—neurons on the banned positions can be excluded from the network. This operation will cause the reduction IN calculating time of network, which is a great advantage—the considered network is a part of real-time system. In the Fig. 6, a graphical interpretation of this restriction can be seen.

Taking all these terms into consideration, the energy function can be expressed in a form of the equation:

18 $$ \begin{aligned} IEF=&-a\sum\nolimits_i\sum\nolimits_k\sum\nolimits_j \sum\nolimits_l C_{ik,jl}v_{ik}v_{vjl}\\ & +b\left(\sum\nolimits_i\sum\nolimits_k\sum\nolimits_{l\neq k} v_{ik}v_{jl}+ \sum\nolimits_i\sum\nolimits_k\sum\nolimits_{j\neq i} v_{ik}v_{jl}\right)\\ & +c\sum\nolimits_i\sum\nolimits_k\sum\nolimits_{l\leq k} v_{ik}v_{(i+1)l} \sigma_{i,i+1}\\ & +d\sum_i\sum_k\sum_{l} v_{ik}v_{(i+1)l}\sigma_{i,i+1} \xi_{ik,jl} \\ \end{aligned}. $$

Having the energy function given as (18), the calculation of interconnection of weights and external currents is possible. To this end, it is necessary to compare the expression of the energy function (18) to hamiltonian function of the Hopfield network (6). The interconnection of weights and external currents is given the following equation:

19 $$ \left\{\begin{array}{ll} t_{ik,jl}= & aC_{ik,jl}-b\delta_{ij}(1-\delta_{kl})-b\delta_{kl}(1-\delta_{ij})\\ &-c\rho_{l<k}\delta_{(i+1)j}\sigma_{i,i+1}-d \delta_{(i+1)j}\sigma_{i,i+1}\xi_{ik,jl} \\ I_{ik}=0\\\end{array} \right., $$

where δ_*ij*_ is Kronecker delta, and sign $\rho_{l<k}$ is defined by the following equation:

20 $$ \rho_{i<k}= \left\{\begin{array}{ll}0&\hbox{ for }\; l>k \\ 1&\hbox{ for}\;l\leq k\\ \end{array} \right.. $$

The values given by (19) are the basis of work performance of continuous Hopfield-like network.

### The maximum neural network

This type of neural structure was introduced by Takefuji and al in [51]. The Maximum Neural Network was defined as discrete Hopfield-like network with the specific activation function: Only the neuron with the highest value of internal potential (in some group) is activated, whereas the rest of the neurons have low potential. The maximum activation function for stereo-matching problem can be formulated as follows:

21 $$ f(u_{ij}) = \left\{\begin{array}{ll} 1&\hbox{if}\quad u_{ij}=max(u_{i1},u_{i2},...,u_{in}) \\ 0&\hbox{otherwise}\\ \end{array}\right. \quad i,j=1,...,n. $$

Taking the discrete nature of this network into consideration, it is not necessary to use the Euler discretization, and internal states of neurons can be calculated by means of the following equation:

22 $$ u_{ik}=\sum_j\sum_l t_{ik,jl}v_{jl}+I_{ik} . $$

This kind of neural network found its applications in optimization problems [64–66]. Unfortunately, in its original form, the maximum network is not fit to solving the stereo-matching problem. The reason is the same as for discrete Hopfield neural network where the stereo-matching problem is too complex and so the network is trapped in local minima. A network in given state may only evolve if any of its neighboring states have a lower energy. Otherwise, the network does not evolve even when there are other further states with lower energy. The evolution is also dependent on the sequence of activated neurons (asynchronous implementation). The smallest possible movement in the state space is the distance between two neighboring vertexes. Thus, starting from the same initial state, possible final states may be very different, following several activations and depending on the sequence of activated neurons. This is the reason why this kind of network is used in association with the classic analog Hopfield-like network. The energy function of Maximum Neural Network used as the component of HMNN is similar to the energy function of the analog Hopfield-like net (18). The only difference is that there is only one term of uniqueness, whereas the second is accomplished by the activation function. Modified expression on interconnection’s strengths was written as (23).

23 $$ \left\{\begin{array}{ll} t_{ik,jl}= & aC_{ik,jl}-b\delta_{kl}(1-\delta_{ij})-c\rho_{l<k}\delta_{(i+1)j}\sigma_{i,i+1}\\ & -d \delta_{(i+1)j}\sigma_{i,i+1}\xi_{ik,jl} \\ I_{ik}=0\\ \end{array}\right.. $$

## Experimental results

**Fig. 7:**
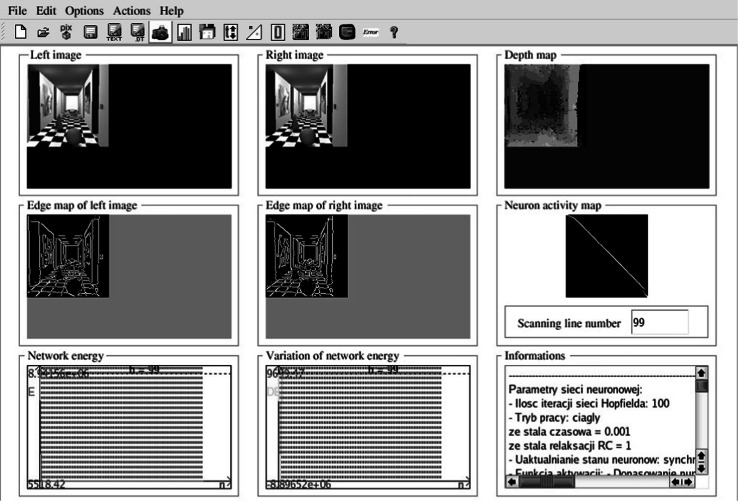
The interface of *Dtest* environment

**Fig. 8:**
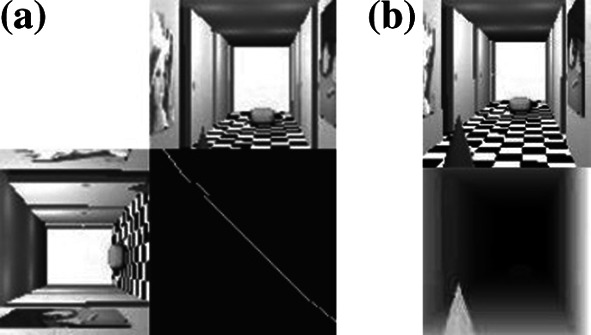
The ideal map of neuron activity with simulated stereo pictures (**a**) and ideal depth map (**b** below) for simulated scene (**b** above)

The proposed method was implemented on a personal computer with Pentium IV –2.80 GHz CPU and 2 GB SDRAM. The stereovision system was tested on *Dtest* environment, written by the author, implemented the use of the C++ language under a Linux environment, as shown in Fig. 7. The neurons activity map is helpful to the analysis of network work performance (can be seen in Fig. 7—middle graphical window on the right). It can be interpreted as a graphical form of fitting matrix to the investigated line—white points mean the neurons with high potentials, and black points correspond to the neurons with low potentials. Intermediate colors correspond to the values between 0 and 1. The neuron activity map is defined for one epipolar line. Thanks to the neurons activity map, the dynamics of neural network can be observed (the map is updated with each iteration). The graphical interpretation of depth map is as follows: The lighter the point on the depth map, the nearer the corresponding point in the scene is. The ideal map of neurons activity for 80 image line (for better understanding shown with stereo images cut at 80 image line) with a simulated picture and the ideal depth map of this picture can be seen in Fig. 8. The resolution of input stereo images is 100 × 100. This resolution is sufficient as far as the imaging of real scene is concerned (each details can be seen), and a complexity of the problem can also be accepted for simulation on PC’s. The author did not find any information in the literature about the pictures used in other simulations of stereovision systems. For these reasons (neural algorithms known from articles and author’s structure), the same images with resolution of 100 × 100 points were used for each test. The images were calibrated in order to find corresponding lines, before starting the stereo-matching procedure. This process allows scanning of pictures line-by-line, which decreases the complexity of the method.

To verify the efficiency of the proposed method, an experiment was performed with the use of both simulated and real images. The use of simulated images enabled the error calculation. The average relative error seems to be the most appropriate. Hopfield-like neural networks work in an indeterministic way, and the solutions, obtained by neural algorithms, can differ from each other. Thanks to the ability of averaging the number of errors for each scan line, this method of error calculation can give the results characterizing the method of work performance. Thanks to the use of simulated images, the expected value for each scene point $\overline{d}_{ik}$ is known. Respecting the percentage notation, the error of stereo-matching process can be written as the following equation:

24 $$ \delta d=\left(\frac{1}{n^2} \sum_i^n \sum_k^n \frac{\|d_{ik}-\overline{d}_{ik}\|}{d_{ik}} \right) 100\,\% . $$

The error is calculated automatically in Dtest program after loading the model depth map (for the whole picture) or after loading the model neuron activity map (for selected image line).

The important parameter of stereovision neural algorithm was the time of working. As a measure of time, the number of epochs was taken. As the point of network’s stabilization was assumed, the iteration in which the energy’s decreasing value was under the value of $\varepsilon$ was determined in tests on artificial images.

**Fig. 9:**
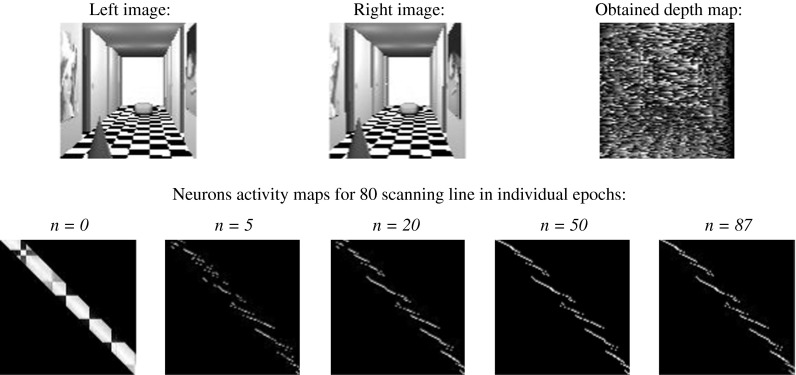
The result of stereo-matching process carried out by discrete Hopfield-like neural network

In order to verify the efficiency of algorithm, worked out by the author, a comparison with solutions known from literature was carried out. Each neural stereovision algorithm was simulated in the same experimental conditions (the same hardware and input images, the same software - Dtest program). The output depth map (the error of depth’s finding) and the time of simulation (the number of epochs) were taken into consideration. Firstly, the discrete Hopfield-like neural network was investigated in stereo-matching problem. Very promising results, shown in [47], encouraged the author to use this structure. The results of simulations for simulated images can be seen in Fig. 9. The author used algorithm and energy function described in [47].

**Fig. 10:**
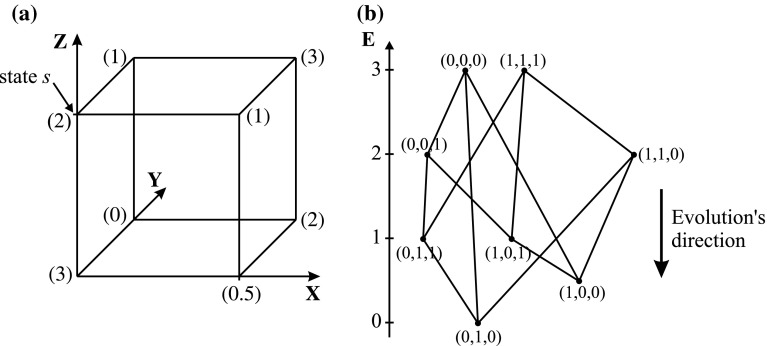
The representation of possible states for a Hopfield neural network with three neurons (**a**), the numbers indicate the energy associated with each state and possible evolution trajectories of this network (**b**)

As can be clearly seen in Fig. 9, the result of stereo-matching process is really poor. The relative error amounts 60,02 %. The time of simulation amounts 87 epochs, but it must be stressed, that as an epoch the time of 10,000 neurons updating is taken (simulation was asynchronous, so it is difficult to say about epochs—one random neuron was updated at the same time). In the author’s opinion, the main problem resides in the mode of network. Binary neural net, used in very complex problems, can behave in an indeterministic way. This is caused by trapping in local minimum of energy [67]. A network in a state *s* may only evolve if any of its *n* neighboring states has a lower energy. Otherwise, the network does not evolve even when there are other further states with lower energy. Only the surface of the solutions hypercube can be penetrated, and there is no possibility of the hypercube’s interior penetration (only discrete values of neuron’s output are possible). The evolution is also dependent on the sequence of activated neurons (asynchronous implementation). The smallest possible movement in the state space is the distance between two neighboring vertexes. Thus, starting from the same initial state, the possible final states may be very different, following several activations and depending on the sequence of activated neurons. The situation is shown in Fig. 10.

Another problem in the above-described structure was the form of energy function. In [47], the authors used the term of uniqueness in the form of following equation:

25 $$ E_2= \sum_i\left(1-\sum_k v_{ik}\right) ^2 +\sum_k\left(1-\sum_i v_{ik}\right) ^2. $$

This form can be appropriate only for analog networks. One can notice that the Eq. (25) in one row or a column generates exactly one neuron in high state of fitting matrix and rest in low state. That indicates that each point for left image must be matched to the points from the right image and inversely. Such situation is of very rare occurrence in the case of stereo-matching problem. It is impossible to have rows or columns with each neuron in low state (which means lack of matching). It is possible only for continuous activated neurons (neurons in low, but non-zero states).

**Fig. 11:**
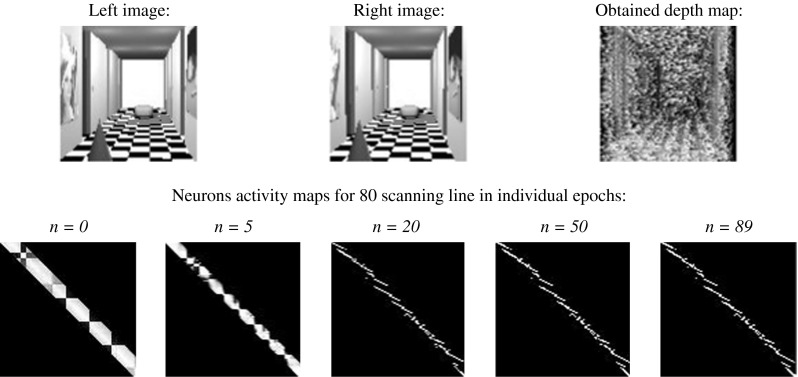
The result of stereo-matching process carried out by discrete Hopfield-like neural network with continuous activation function

Then, t the discreet network, but with continuous activity function, was investigated. The procedure and the energy function were taken from [48]. The results of the simulation can be seen in Fig. 11.

As can be clearly seen, the result of simulation is not much better than in the case of the discrete-time Hopfield network with discrete activation function. The relative error amounts 59,62 %. The time of simulation amounts to 89 epochs. As can be concluded, the main problem is discrete architecture of network, not the form of activation function. In this case (continuous activation function), the form of the term of uniqueness in the form (25) can be accepted.

**Fig. 12:**
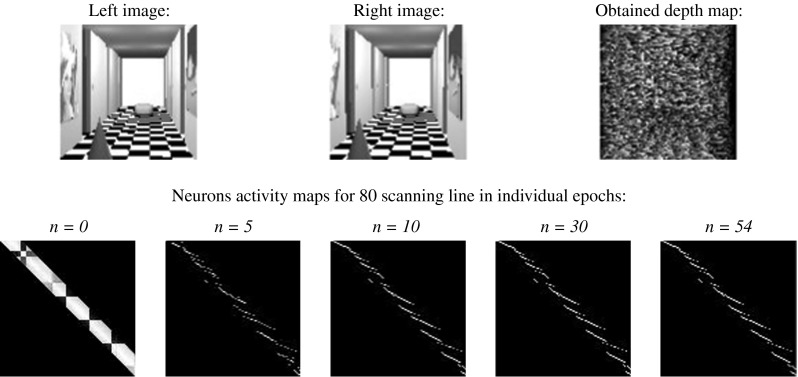
The result of stereo-matching process carried out by maximum neural network

The maximum neural network had not t used in stereo-matching problem before. But it must be stressed that maximum network is a kind of discrete Hopfield-like network with specified discrete activation function, so it can be predictable that this kind of network cannot give better results than in previous cases. The result of simulation of this kind of structure can be seen in Fig. 12.

Each comment concerning the discrete network which was first tested is legitimating to maximum network. The relative error amounts to 68.52 %. The only advantage was the time of simulations: 54 epochs.

**Fig. 13:**
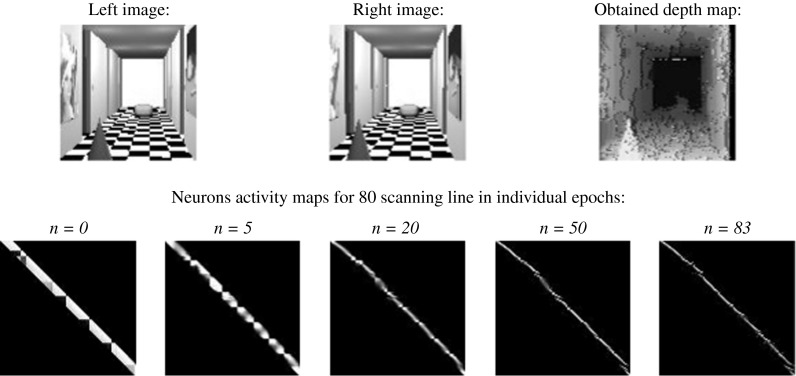
The result of stereo-matching process carried out by continuous Hopfield-like neural network with a sigmoidal activation function

The most appropriate solution to the stereo-matching problem seems to be the analog neural network, described in [44–46]. The author used his own energy function 18 to algorithm. The results of such simulation can be seen in Fig. 13.

As can be seen, the result is much better than in the previous cases. The relative error amounts to 19.89 %. It is much less, than for the previous network. The only problem is the computing speed—83 epochs. The author tried to decrease this time without losing the accuracy by means of the new architecture of network—HMNN.

An operational procedure for solving the stereo-matching problem by HMNN is summarized as follows:

1. Assume number of image epipolar line *h* = 0;
2. Assume maximum number of iteration *it*_max_ enough to find attraction area of global minimum;
3. IEF mapping into the analog Hopfield network:
  - Compute external inputs of neurons and their interconnection strength using (19) (with the upkeep of symmetrical interconnection strength’s matrix);
  - Initialize states of neurons in heuristic way—assume potentials *v*_*ik*_ proportional to correlation coefficients *C*_*ik*_;
4. Continuous Hopfield network updating procedure for energy minimization (working in continuous Hopfield mode):
  - For each neuron, compute the internal potential, with the use of (8);
  - For each neuron, compute the external potential using (5);
  - If number of iteration is equal *it*_max_, go to (5), else go to (a);
5. IEF mapping into Maximum Network:
  - Compute external inputs of neurons and their interconnection strength using (23) (with keeping of symmetrical interconnection strength’s matrix);
  - Assume states of neurons the same as at the end of working of continuous Hopfield network;
6. Maximum Network updating procedure for energy minimization (working in maximum mode):
  - For each neuron, compute the internal potential, with the use of (22);
  - For each neuron, compute the external potential using (21);
  - If changes of internal potentials for each neurons equal zero, proceed to (7), otherwise, proceed to (a);
7. If present epipolar line is not the last one, increment number of line *h* = *h* + 1 and proceed to (2), otherwise, proceed o to (8);
8. End simulation.

It was assumed that the attraction area of the global minimum was usually reached after 50 iterations (empirically confirmed). In maximum mode, a stable state is reached after at most 20 iterations, and this limit of iteration was assumed in order to have possibility of confirmation of results reached for different stereo images.

**Fig. 14:**
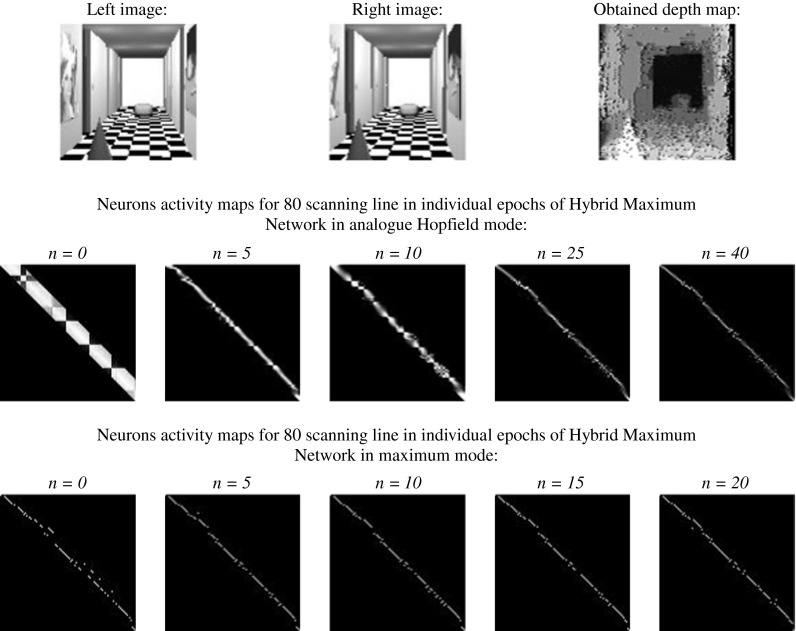
The result of stereo-matching process carried out by HMNN for simulated stereo images

**Fig. 15:**
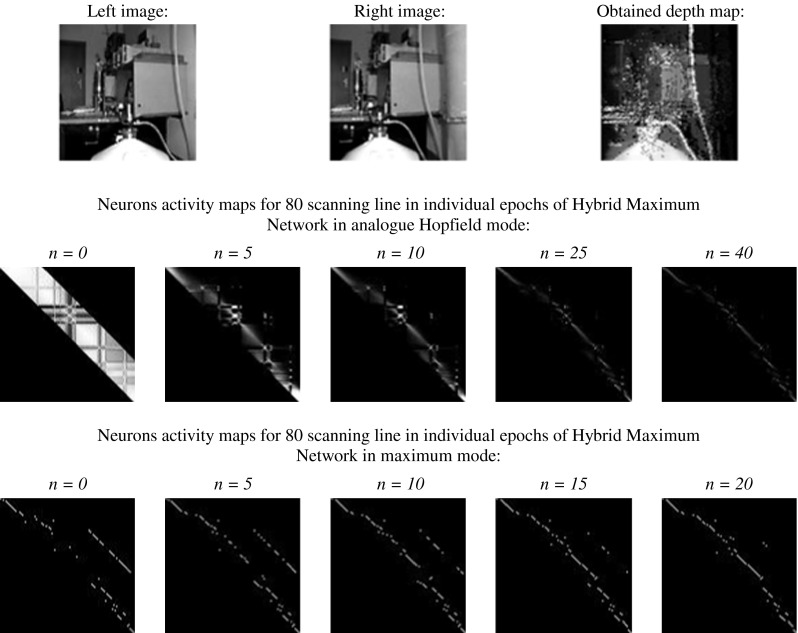
The result of stereo-matching process carried out by HMNN for real stereo images

The results of stereo-matching process, carried out by HMNN, can be seen below, for simulated images in Fig. 14 and for real images in Fig. 15.

At Figs. 14 and 15 in the first row, stereo pictures, used for stereo-matching process, were shown. The second row shows neurons activity maps for 80 scanning line (arbitrary assumed) in iterations (number of "*n*") of net working in analog Hopfield mode. In the next row, the obtained depth map can be seen. The same sequence was repeated for the network working in maximum mode.

The error of stereo-matching process can be calculated only for simulated pictures (possibility of neurons activity map determination).

The analysis of network working (Figs. 14, 15) shows that in analog Hopfield mode, fitting is non-uniqueness. This can be concluded by analyzing the neurons activity maps. In the case of uniqueness, stereo matching in each row and each column of fitting matrix (its graphical form is neurons activity map) should be placed at very most one non-zero element, whereas in stable state, few non-vanishing elements can be observed in columns and rows of fitting matrix. Because of non-uniqueness, it is difficult to say anything about the sequences in the areas. Also, the term of depth continuity upkeep in areas cannot be stated. This mistake can be corrected in maximum working mode. Maximum activation function involves the meeting of uniqueness term, which can be seen in iterations of network in maximum mode. In each line of FM, at the most one non-zero element can be seen. Stable state is reached very fast thanks to limitation of possible neuron states' configuration (maximum activation function). Compared with the analog Hopfield network, the Hybrid-Maximum Network is working with the same efficiency, but much faster.

**Fig. 16:**
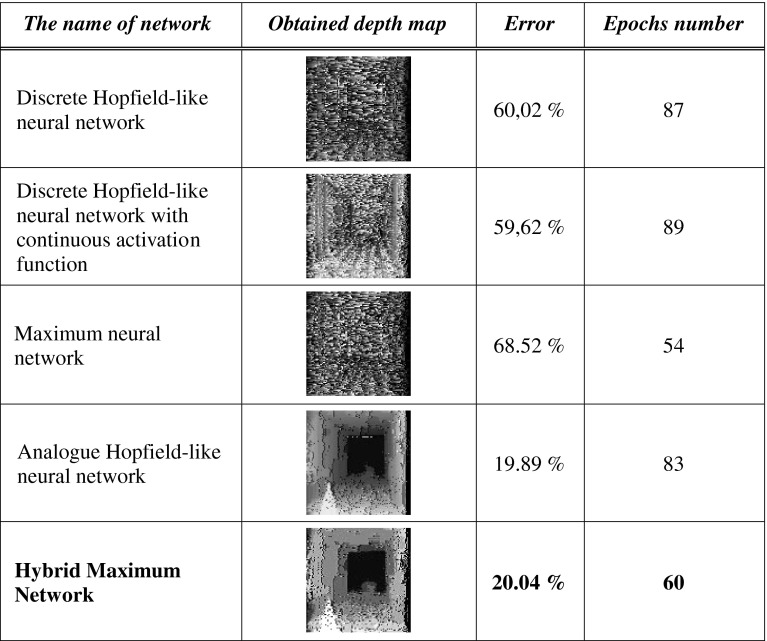
The evaluation of results of working neural networks investigated in the present publication

The results of the Hybrid-Maximum Network were presented side to side with the results of stereo-matching process, performed by some other Hopfield-based networks and mentioned in the present publication. The evaluation of obtained depths, errors, and numbers of epochs needed to reaching stable was depicted in Fig. 16.

As can be clearly seen, it is only the results obtained by continuous and Hybrid-Maximum Networks that can be accepted. Almost the same errors were obtained for this network, but the HMNN was faster—the number of epochs for this kind of structure amounted to 0.72 of analog network’s epochs. The decrease in time of work performance is very important, as far as the use in real time is concerned. Taking this into consideration, it must be said that HMNN is working in the most efficient way among each investigated neural structures.

## Conclusion

This study shows the use of an innovative architecture of Hopfield based on neural network–Hybrid-Maximum Network. The network introduced here has been used in stereo-matching process. The stereo correspondence problem has been formulated as an optimization task where an energy function of network which represents the mapping of all constraints of the solution, is minimized. The advantage of using a Hopfield neural network is that a global match is automatically achieved because all the neurons are interconnected in a feedback loop so that the output of one affects the input of all the others. The convergence into a stable state is guaranteed for continuous Hopfield-like network with continuous activation function. The parallel execution capability of this structure is also a powerful property that should be taken into consideration in terms of the target system assisting aged and/or visually impaired people. Additionally, thanks to the use of maximum mode, the time of computation significantly decreases.

The experimental results indicate significant gains from using of maximum mode after finding the global minimum’s attraction area. A comparative analysis, performed with the classical Hopfield network (analog and discrete), Maximum Network and Hybrid-Maximum Network, indicated a better performance of latter type of network. The solution to the stereo correspondence problem was similar to that obtained by the analog Hopfield-like network, but the number of iterations was much smaller

The computational time is crucial to real-time applications . Thus, the use of novel Hybrid-Maximal Network is justified.
